# Using Black Hole Algorithm to Improve EEG-Based Emotion Recognition

**DOI:** 10.1155/2018/3050214

**Published:** 2018-06-11

**Authors:** Roberto Munoz, Rodrigo Olivares, Carla Taramasco, Rodolfo Villarroel, Ricardo Soto, Thiago S. Barcelos, Erick Merino, María Francisca Alonso-Sánchez

**Affiliations:** ^1^Escuela de Ingeniería Civil Informática, Universidad de Valparaíso, Valparaíso, Chile; ^2^Pontificia Universidad Católica de Valparaíso, Valparaíso, Chile; ^3^Instituto Federal de Educação, Ciência e Tecnologia de São Paulo, Brazil; ^4^Centro de Investigación del Desarrollo en Cognición y Lenguaje, Universidad de Valparaíso, Valparaíso, Chile

## Abstract

Emotions are a critical aspect of human behavior. One widely used technique for research in emotion measurement is based on the use of EEG signals. In general terms, the first step of signal processing is the elimination of noise, which can be done in manual or automatic terms. The next step is determining the feature vector using, for example, entropy calculation and its variations to generate a classification model. It is possible to use this approach to classify theoretical models such as the Circumplex model. This model proposes that emotions are distributed in a two-dimensional circular space. However, methods to determine the feature vector are highly susceptible to noise that may exist in the signal. In this article, a new method to adjust the classifier is proposed using metaheuristics based on the black hole algorithm. The method is aimed at obtaining results similar to those obtained with manual noise elimination methods. In order to evaluate the proposed method, the MAHNOB HCI Tagging Database was used. Results show that using the black hole algorithm to optimize the feature vector of the Support Vector Machine we obtained an accuracy of 92.56% over 30 executions.

## 1. Introduction

Emotions play an important role regarding the way in which people think and behave [1]. In physiological terms, emotions are phenomena of short duration that represent efficient modes of adaptation to the constant demands presented by our environment [2]. One of the most accepted models that represent emotions is known as the Circumplex model [3]. This model organizes emotions into points on a bidimensional plane made up of the following dimensions: “Valence” (pleasurable or not pleasurable) and “Arousal” (tension, relaxation); in this way, emotions are organized in a circular manner within this plane [3]. Furthermore, different methods exist for measuring emotions within people; those with the most precision are based on electrophysiological signals, which can be captured, for example, by an electroencephalogram (EEG) device.

In particular, the increase of the visual component P1 has been studied, with event-related potentials (ERP), by filtering low spatial frequencies, thus evidencing the rapid activation of the magnocellular system against stimuli that trigger emotions of high agitation [4]. The component P1 is of early onset and precedes facial recognition; therefore, it is possible to estimate that emotional processing manages to circumvent the track of regular visual processing when emotions contained in the stimuli are of high agitation [5]. Furthermore, by means of a classifier generated from the combination of wavelet entropy and the averaging of wavelets of EEG signals associated with emotions, a valence of 76.8% and an agitation of 74.3% have been recognized. Furthermore, the decoding of pleasurable or unpleasant emotions known as valence [6] has been obtained through Linear Discriminant Analysis. In this context and considering both the temporal resolution of EEG signals and the possibility of applying ecological tasks to subjects while registering signals, classification methods are a tool of great potential for the study of emotions.

One of the metrics (features) that is the most representative and which provides the most information is entropy. Entropy is a measurement of information or order; it measures the predictability of data. This is, given a set of *N* data elements, *X* = 〈*x*_1_, *x*_2_,…, *x*_*N*_〉, entropy is the probability of being able to predict an element *x*_*i*_, i.e., the homogeneity or heterogeneity of the data.

However, this use of entropy can magnify the signal noise, being extremely sensitive to minimal variations. For this reason, different ways of measuring entropy have been proposed, such as approximate entropy, differential entropy, or sample entropy. Among these methods, sample entropy presents a valuable statistical consistency and for this reason was utilized as a basis of comparison [7]. Sample entropy (SampEn) is based on approximate entropy, which by means of elimination of repeated information prevents the evaluation of indeterminate logarithms and self-matching, which can result in inconsistent and erroneous data and thus also achieve a greater statistical consistency.

Although the SampEn method is highly accurate, it is extremely sensitive to its input parameters. In fact, there is no established consensus on the selection of parameters for small data sets, especially for biological data [8]. Another problem in the calculation of SampEn is that if the sampling space is not significant, the built classifiers can produce values with high levels of error.

These situations present the problem of finding or calculating the most suitable value for entropy that allows generating high performance classifiers. This task is complex and can be seen as an optimization problem in itself. A first approximation to a potential solution can be the use of full-search algorithms to explore a tree of extremely large potential solutions. However, these techniques are highly costly and can lead to an unsuitable large amount of attempts to find a solution. With this is mind, it is not possible to propose complete techniques such as Backtracking or hybrid ones such as Forward Checking.

On the other hand, recently, several approaches have emerged, inspired by natural phenomena, that allow solving complex optimization and combinatorial problems in reduced time periods [9–12]. These techniques have been successful when the complexity of the problem is not linear, given that they do not explore the solution tree in their completeness.

In this article, we propose using an approximate optimization approach to find the best values considering the predictability of the classifier. The reason for the proposed approach is the strong impact on the development of classifiers for emotion recognition based on electroencephalography. The main idea is to use the black hole algorithm due to its low cost, similar to the calculation of entropy. This algorithm is inspired by the phenomena of black holes [13] and will be used to build the classifier iteratively. This method will improve and update the classifier according to its level of performance: lower percentage of error will be associated with better evaluation levels.

The present work is organized as follows: Theoretical background is introduced in Section 2. In Section 3, we detail the required resources to apply our approximation approach.  Section 4 illustrates the computational experiments including a comparison with the results obtained using the traditional calculation method. Finally, conclusions and future works are described in Section 5.

## 2. Background

First, in Section 2.1 we present the theoretical model for the classifications proposed by Russell that supports our work [3]. In Section 2.2, we describe some components associated with electroencephalography and their relationships with emotions detection. Following, in Section 2.3, we present the sample entropy, which is an alternative to entropy. This method is the main component of the feature vector which is classified by a Support Vector Machine (SVM). This model is composed by a set of supervised learning algorithms and they are described in Section 2.4. In Section 2.5 we expose some techniques used for the EEG signals treatment. Finally, in Section 2.6 we will present two relevant works detecting emotions with EEG.

To conclude, our proposal consists of the preprocessing of the signal (through EMD and sample entropy) for the construction of an initial multiclass SVM classifier. Using this classifier as a base, a population (group) of classifiers is created, which are formed by groups of modified characteristics coming from the initial characteristics and a random variation relative to the error of the classifier.

**Figure 1:**
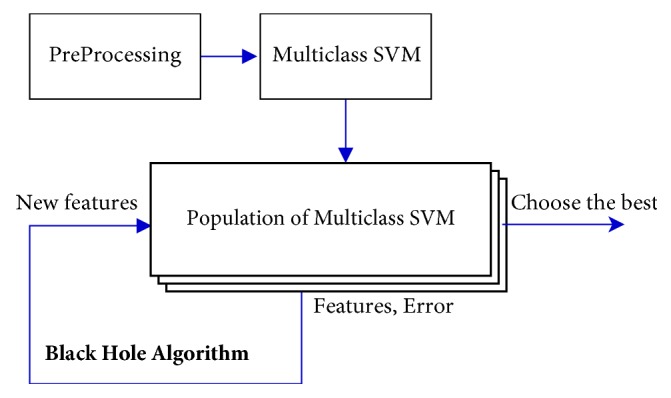
Proposed approach.

Once this population is created, it is iterated through the black hole metaheuristic, which continuously generates and improves these characteristics in order to obtain distinct classifiers; these classifiers are then evaluated, always, using the original characteristics from the signal. Once all the iterations are completed, the best classifier (historically speaking) is chosen; this classifier is, finally, utilized.  Figure 1 shows a scheme of our proposal.

### 2.1. Theoretical Model for Emotion Classification

**Figure 2:**
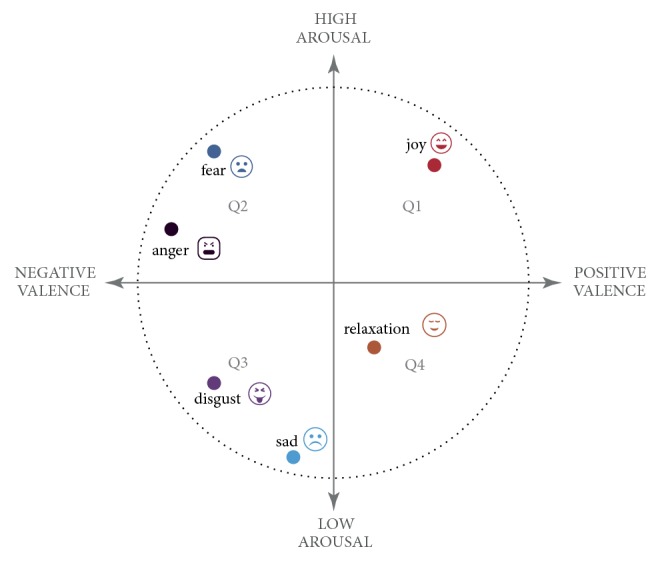
Russell's classification model [3, 14].

Circumplex is one of the most used models for emotion classification [3]. This model is composed by two dimensions. One dimension is known as the valence dimension, which varies from “negative valence” to “positive valence”. The second dimension is called arousal, which varies from “low arousal” to “high arousal”. A graphical representation of the Circumplex model is presented in Figure 2.

There are variants of the Circumplex model in which extra dimensions are added, such as domination or freedom in a given situation [3]. However, it has been proven that this dimension captures the believed consequences by the person regarding emotion and not the emotion itself [14].

In this work, we have used a discrete quadrant division to represent the greatest variation among emotional states. This approach is optimal for classifying and obtaining fewer error rates. This is because they represent the greatest possible distance between agitation and valence (the digital axes). On the other hand, it would be possible to classify discrete emotions; there would be a greater probability of erroneously classifying nearby emotions in this model because they would represent lower variance values.

### 2.2. Electroencephalography

Electroencephalography is a method of neurophysiological exploration that is based on the registry of cerebral activity through sensors that translate bioelectric activity into electrical current [15]. It is a noninvasive method that allows the measurement of voltage fluctuations that result from the ionic current of the postsynaptic potentials of neurons.

EEG signals are usually classified by their frequency, amplitude, shape, or electrode position. The EEG bands are *δ* (lower than 4Hz), *θ* (between 4Hz and 7Hz), *α* (8-15Hz), *β* (16-31Hz), *γ* (higher than 31Hz), and *μ* (between 8 and 12 Hz). These bands describe several emotional states [16], although there are alternative definitions for the bands. For example, the Beta Band frequency range may begin at 12, 13, 14, or even 16 Hz as described in [17], where the *mu* band is not even defined.

**Figure 3:**
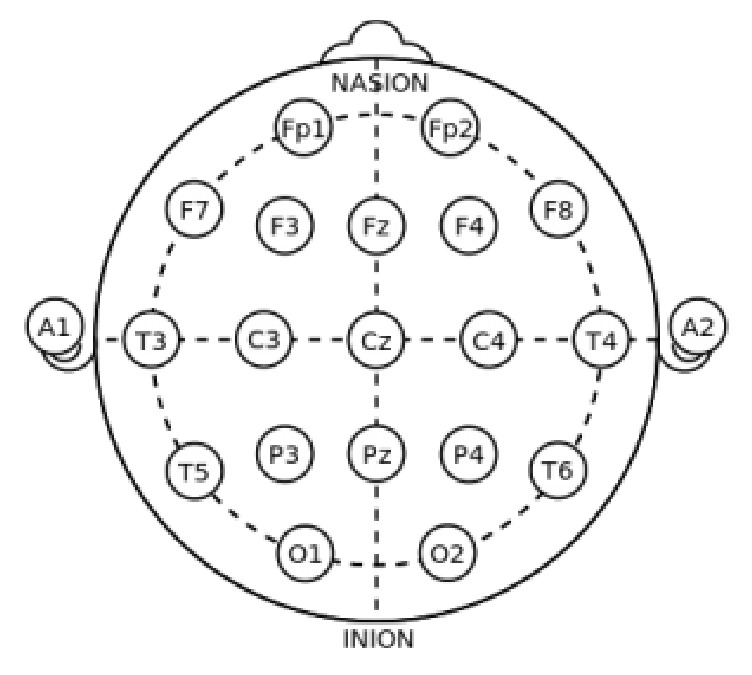
EEG 10-20 system.

Even so, the position of sensors is standardized by the 10-20 channel system, by which each position is described by a combination of a letter and a number. The letter indicates the brain region that may be represented as frontal (F), central (C), temporal (T), occipital (O), or parietal (P) [18]. Even numbers indicate positions at the right side of the brain, while odd numbers indicate positions at the left side. The system name refers to the use of 10% and 20% proportions to position the electrodes in relation to four cardinal points: ears, nape and nasion [19] (as shown in Figure 3).

There is also another positioning system named 10-10 system in which only the 10% proportion is used. In this alternative system the same bands mentioned before are used with the addition of other intermediate channels. In the case of the lobes, letter combinations are created for the channels between two regions, for example, FP for frontoparietal [20].

The assembly of the electrodes can be done by referencing the electrodes or with a bipole method. The reference is made with electrodes that generate a comparison link, generally with an electrode positioned in A2 (the ear electrode) and the bipole method is performed by recording the potential differences between paired electrodes [21].

The applications of EEG are varied [22–26]. However, its most known use is for clinical diagnosis [27]. In recent years, however, its use has spread in the research of brain functions associated with cognitive processes. One of the most commonly used techniques is event-related potentials (ERP) that allows the repeated measurement of ongoing brain activity segments immediately after the presentation of a stimulus. In this way, by averaging the segments it is possible to measure the cerebral voltage associate with the stimuli presented; i.e., by means of an analysis of time amplitude, it is possible to associate components to the stimuli [28]. It is also possible to analyze the oscillations related to events in the frequency domain. This analysis can be performed in the frequency domain with the analysis of the spectral decomposition represented in power spectral density of each trial through the Fourier transform. However, the time variable with a Fourier transform applied to a series of consecutive time windows or with a discrete Wavelet Transform Analysis can be included. The so-called rhythms have gained popularity in the research of social neuroscience and frequency bands (i.e., alpha) have been associated with cognitive processes and mental states. Because of this and because of the particular suitability for the investigation of emotions, this study focused on frequency analysis.

### 2.3. Sample Entropy

Sample entropy is a variation of approximate entropy (ApEn). This entropy reduces the potential bias generated by self-matching that arises during ApEn [29]. The function of SampEn is the negative of the natural logarithm of the conditional probability that two similar sequences, with a distance of less than *r*, for *m* points, continue to be so when increasing the number of points from *m* to (*m* + 1). This is to say that the SampEn is calculated by

(1) $$\begin{array}{c} SampEn\left(\;m,r,N\;\right)=-\ln\left(\;\frac{C_{m+1}\left(r\right)}{C_{m}\left(r\right)}\;\right) \end{array}$$

where *C*_*m*_ is defined as

(2) $$\begin{array}{c} C_{m}\left(\;r\;\right)=\frac{\left\{\text{number of all probable pairs }\left(i,j\right)\text{ with }\left|x_{i}^{m}-x_{j}^{m}\right|<r,\;i\neq j\right\}}{\left\{\text{number of all probable pairs, }i.e.\left(N-m+1\right)\left(N-m\right)\right\}} \end{array}$$

where |*x*_*i*_^*m*^ − *x*_*j*_^*m*^| denotes the distance between the points *x*_*i*_^*m*^ and *x*_*j*_^*m*^ in the dimension space to be evaluated, *m*. The variable *r* represents the tolerable standard deviation of the time series. Furthermore, *N* represents the length of the time series. Finally, it has been shown that SampEn has a better statistical validity for *m* = 1 or 2 and the range of *r* in the interval between 0.1 and 0.25.

### 2.4. Support Vector Machines

Support Vector Machines (SVM) are a set of supervised learning algorithms based on statistics learning theory [30]. SVMs put all features in n-dimensional space (the number of dimensions of the feature vector, 8 in this case) and adjust them to a defined kernel space (Gaussian, polynomial, etc.). To build a multiclass SVM, we use the one-against-all method. This technique consists of constructing *k∗*(*k* − 1)/2 binary classifiers (hyperplanes), separating each class from another, and applying a voting system [31].

The main advantage of using SVMs is that their model can be generalized for nonlinear feature spaces. On the other hand, weighted SVM, which is the method used in this work, has a regularization parameter C that enables accommodation to outliers and allows errors on the training set.

### 2.5. Signal Processing Algorithms

#### 2.5.1. Technique: Empirical Mode Decomposition

Empirical Mode Decomposition (EMD) is a data-driven signal processing and analysis technique [32]. This technique breaks down the signal into its basic components, similar to the creation of harmonics (fundamental sinusoidal), but with the advantage that each signal has frequencies and variable amplitudes, obtaining more information in each component [33].

The main advantage of using this technique is that it permits softening the signals and decreasing noise, which is especially useful in physiological signals.

Each component fulfills 2 fundamental requirements:

- The number of endpoints and the number of crosses by zero (zero-crossings) is equal or differs at the most in 1.
- The average between the top and bottom wrapper is always zero at each point.

EMD generates a set of Intrinsic Mode Functions (IMF) that allows obtaining the components of a signal with most significance. The steps to define the set of functions are as follows:

(1) Identify all of the local endpoints of the signal.
(2) Connect all local maximums using cubic spline interpolation to create a superior wrapper.
(3) Repeat the same process for the local minimums.
(4) Create a *m*[*x*] signal, which is the average of both wrappers.
(5) The first resulting signal is the original signal minus *m* (average) signal:
  (3) $$\begin{array}{c} IMF\left[\;n\;\right]=x\left[\;n\;\right]-m\left[\;n\;\right] \end{array}$$
(6) The remainder of the original signal is obtained minus the IMF; i.e.,
  (4) $$\begin{array}{c} r\left[\;n\;\right]=x\left[\;n\;\right]-IMF_{1}\left[\;n\;\right] \end{array}$$
(7) If IMF satisfies the definition (the 2 basic requirements), it is accepted as a valid IMF; otherwise the process is rejected and repeated using the remainder as the original signal.

This continues until the stopping condition is met, which can be a certain number of iterations or until the residue contains no more than one endpoint.

#### 2.5.2. Technique: Wavelet Transform

The use of wavelet transformation for EEG signal classification was proposed by [34]. To do this, the signal is decomposed in a set of basic signals called wavelets. These signals are obtained from a mother wavelet, which is a signal wavelet prototype that was generated through dilatations, contractions, and signal changes. The wavelet coefficients resulting from this analysis represent similarity between the scaled/shifted wavelets and the original data. Despite the fact that this method of analysis permits obtaining a higher temporal resolution than the Fourier transform, the frequency resolution is lower in the low frequencies. Also, in the high frequencies, when the frequency resolution increases, the temporal resolution decreases.

In spite of the mentioned limitations, the frequency analysis of wavelet has been used, among other things, to determine the intracortical coupling, unraveling cerebral synchrony through the systems of communication between near and distant neurons associated with cognitive processes [35]. Likewise, the analysis of oscillations has been relevant in the study of mirror neurons, which, according to some authors, is the basis of empathy [36]. The rhythm Mu (*μ*) (8-12Hz) in the sensorimotor cortex, associated with the system of mirror neurons, is more active when subjects are at rest and it is desynchronized when an action is carried out or an action is observed [37]. In this way, the study of the synchronization of the oscillations has been of great importance for the understanding of aspects such as empathy, emotional reactions, and even social interactions [38].

#### 2.5.3. Comparison: EMD versus Wavelet

EMD is an iterative process that allows a transversal time-frequency analysis by extracting the oscillatory characteristics. On the other hand, the wavelet transform allows performing a longitudinal analysis of the frequency changes over time by convolving a signal based on a mother wavelet. Particularly the EEG signals are characterized by being non-Gaussian and nonstationary; due to this, it has been observed that the wavelet transform has a worst resolution of time and frequency while the EMD provides a more intuitive understanding of the data [39]. In addition, the EMD does not have the need for arbitrary bandpass filter cut-offs and the phase is detected independent of the amplitude.

### 2.6. Relevant Works

#### 2.6.1. Applications: WEAVE Algorithm

WEAVE is EEG-emotion valence classifier based on five steps:

1. Segmentation of EEG signals related to emotions in windows of 6 seconds.
2. Extraction of the wavelet metrics to form WEAVE.
3. Calculating the complexity of metrics with Normalized Mutual Information (NMI) [40].
4. Reduction of channels through NMI.
5. Classification with the Support Vector Machine (SVM) algorithm using the Sequential Minimal Optimization (SMO) algorithm to train the SVM.

The advantages of the wavelet transform are due to the regularity in the intersegment estimation and the subbands obtainment through the bandpass filter and the denoiser signal decomposition [41].

#### 2.6.2. EEG-Based Emotion Recognition Using Combined Feature Extraction Method

A state of excitement in the cerebral cortex can be identified using the detection of a significant Beta Band [42]. This state is recognized as a favorable scenario for emotion recognition [43, 44].

In [42], a method is proposed for the recognition of emotions using Empirical Mode Decomposition (EMD) and the sampled entropy for the generation of a classifier using SVM. The main advantage of this method is that only 2 channels are used (F3 and C4). EMD is used on both signals to calculate the first 4 Intrinsic Mode Functions (IMFs). Each of the 8 resulting IMFs is calculated with SampEn. Later, this entropy is used for the characteristics vectors and to be entered into the SVM for training and testing.

For the reconstruction of the Beta Band they used low pass and high pass Butterworth filters. Signals were filtered using a 3rd-order bandpass Butterworth filter [45] with a cut-off frequency of 12.5 and 30 Hz and the resonant frequency equal to 0.1 Hz [46].

**Figure 4:**
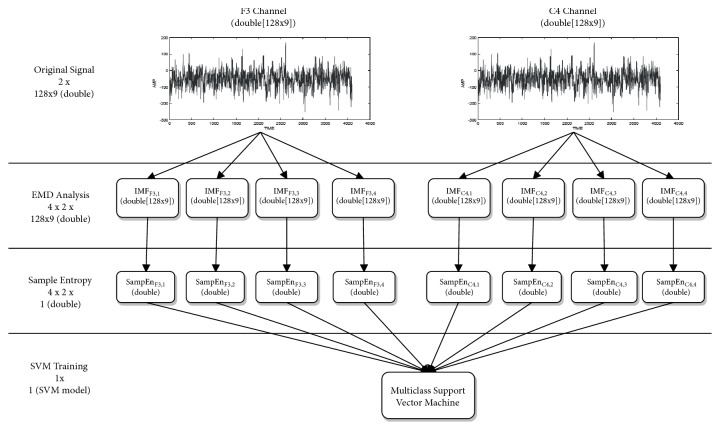
EEG-based emotion recognition using combined feature extraction method.

Furthermore, for the experiment, the Database for Emotion Analysis using Physiological Signals (DEAP) was used [47]. In general terms, the experimental results presented by the authors indicate that the proposed method obtains an accuracy of 94.98% for binary-class task and the best accuracy achieves 93.20% for the multiclass task using DEAP database. In this way, the results presented by the authors are highly appropriate in relation to other means of classification. In the Figure 4, we present a working schema of the proposed by [42].

Upon analyzing, in detail, the process, we can see that the entropy values strongly affect the creation of the classifier and are directly related to the configuration of the input parameters. In addition, due to the search process is an iterative procedure, it is not possible to determine the performance of the classifier until the process is finished.

## 3. Materials and Methods

### 3.1. Dataset

For our proposal, presented in Section 3.3, we used the MAHNOB HCI Tagging Database [48]. This dataset is formed by 563 sessions realized by 30 participants. Each session contains data from only one person. Participants were presented with movies and images with emotional content. While they were being presented with the emotional content, they were monitored with EEG of 32 channels, 6 cameras, a microphone in the head (head-worn microphone), an eye gaze tracker, and conductivity, among other sensors.

Furthermore, for each session, participants were asked to answer a survey regarding emotions they felt, levels of agitation, valence and domination, among other questions. We used the agitation and valence (high, low) to create the multilabelled classifier, where each of the four classes is one of the quadrants. When using a multiclass model for classification, the answer must be in one of the classes contained in the model. To avoid the creation of a null class, it is advisable to use the full spectrum of emotions. For this, the Russell quadrant model was selected [3], which includes all the possible emotions discretized in points.

For this study, we used the F3 and C4 channels of the EEG sensor, as it was done in [42]. These channels represent part of the Beta Band, which is significant when the brain is in excited states [49], an ideal condition for recognizing emotions. The activity of the Beta Band is clearer in the frontal, temporal, and central areas, in regions such as F3, F4, C3, C4, T3, and T4. For the selection of channels, a reconstruction of the Beta Band was performed, and the power spectral density (PSD) was calculated. Since the average of PSD in the F3 and C4 was more significant, these were chosen for the realization of this study.

### 3.2. Approximate Methods

In optimization new approximate techniques have been proposed in order to improve the search process. Many of these algorithms are on inspired in social environments, natural phenomena, and the biological evolution [50]. These methods have widely been used to solve uncountable optimization problems [51]. Swarm intelligence is a particular case of metaheuristics that groups a subset of algorithms and it allows solving optimization problems using collective intelligence. For instance, social situations and human behavior have inspired the imperialist competitive algorithm [52] and the brainstorming algorithm [53], respectively. Techniques based on single-solution such as the intelligence water drop algorithm [54] have been proposed. Moreover, approximate methods such as the ant colony optimization algorithm [55] are population-based using the collective intelligence of individuals. On the other hand, techniques inspired by the collaborative behavior of some animals have been proposed in [56–59], among others. More sophistic techniques are inspired by spatial phenomena such as the gravitational search algorithm [60], the black hole algorithm [61], the big bang algorithm [62], and the big bang-big crunch algorithm [63] and others. Finally, genetic algorithms [64] and differential evolution [65] are two of the best-known techniques inspired by the process of natural selection.

### 3.3. Proposed Approach

To solve this problem, we propose to use an approximate method that permits evaluating previous behavior of the classifier, and if necessary, allowing for improvement. The approximate techniques have been widely used in real world problems [66, 67], being very useful when the search space is extremely large and the use of complete search algorithms is unfeasible. While there are many alternatives to solve this problem, we have decided using the black hole algorithm due to the fact that it is relatively easy to implement, and it is slight free from tuning parameter issues. Moreover, this method uses a technique of exploration/exploitation free of external components reducing the probability of being affected to unexpected changes. Finally, as reported in [68], the black hole algorithm in optimization problem converges to global optimal in each evaluation while its competitors' genetic algorithm, ant colony optimization, and simulated annealing can be caught in local optimum solutions.

The black hole algorithm is based on the phenomenon of the same name, which occurs in outer space and is inspired by the law of attraction/absorption. The algorithm follows three main fundamentals:

(1) A star in space is considered a solution to the problem. As a population-based algorithm, a certain number of stars are randomly generated.
(2) The black hole is selected. A black hole represents the star with the best performance of all solutions.
(3) The movement and generation of new stars are carried out through the absorption formula:
  (5) $$\begin{array}{c} x_{i}^{d}\left(\;t+1\;\right)=x_{i}^{d}\left(\;t\;\right)+\alpha \left[\;x_{bh}^{d}-x_{i}^{d}\left(\;t\;\right)\;\right],\;\forall i\in \left\{1,\ldots ,n\right\} \end{array}$$

where *x*_*i*_^*d*^(*t*) corresponds to the *d*th component of the *i*th star in the iteration *t*, *x*_*bh*_^*d*^ is the *d*th component of the black hole in the search space, *n* represents the number of solutions (number of stars), and *α* is a random uniform number of distribution between zero and one. Finally, *x*_*i*_^*d*^(*t* + 1) corresponds to the *d*th component of the location of the *i*th star in the next iteration.

The event horizon is a radius originated by the black hole. In case a star crosses the horizon, it will be absorbed and destroyed by the black hole and a new star (solution) is created randomly. This is known as the probability of crossing the event horizon and is calculated as follows:

(6) $$\begin{array}{c} R=\frac{f_{bh}}{\sum_{i=1}^{n}f_{i}} \end{array}$$

where *f*_*bh*_ is the performance value that has the best solution, *f*_*i*_ is the value associated with the quality of the *i*th star, and *n* is the number of stars (solutions). When the distance between the black hole and the star is less than the radius then the star crosses the event horizon. This star is absorbed and a new is randomly generated. We highlight the variability offered by event horizon that allows resolving the common and complex problem of stagnation in local optimum.

One of the most interesting characteristics of incomplete data processing algorithms is the approximation to good solutions. This concept may be used as stop criteria. However, in situations where the optimal solutions are not known a priori, it is not possible to measure the quality of found solutions. In these cases, possible stop criteria are the number of executed iterations, for the sake of clarity of the proposed algorithm. In our proposal, the stop criteria are initially set as 100 off-line iterations.

Algorithm 1 displays the optimization procedure. At the beginning, the initial *n*-star population is randomly generated for each of the intrinsic signals and loop statement begins working.

Randomness allows a degree of variability in the algorithm. Then, in the loop statement, the process of absorption of the algorithm is carried out. The quality of each solution is calculated, determined by the performance exhibited by the classifier. If the rating value is close to 1, the solution is considered to have a high quality (see Line (11) of Algorithm 1). Conversely, if the rating value is close to 0, the solution is considered to exhibit low quality due to the probability of crossing the event horizon is highest. The solutions are generated by the absorption of stars by the black hole that is presented in (5). Performing this process generates a real number of predictability for each intrinsic signal. If a star or solution reaches a value better than the black hole, its locations are swapped. If a star crosses the event horizon of the black hole, calculated by (6), it is absorbed and generates a new one randomly. This comparison is performed according to a random variable with uniform distribution *r* ~ [0,1]. This whole procedure is done iteratively.

To measure the performance (quality) of the solutions, a proportion given by (6) is used between the fitness of the star and the combined value of all fitness (excluding that star). This value is known as an event horizon. If this percentage value is less than *r*, randomly generated, the star will be absorbed. This nondeterministic process provides variability to the solutions.

Finally, the loop statement ends when an adequate enough solution is reached for our approach; this condition is determined by updating the solution in a certain amount of iterations. At the end, the best solutions are memorized and visualized.

**Figure 5:**
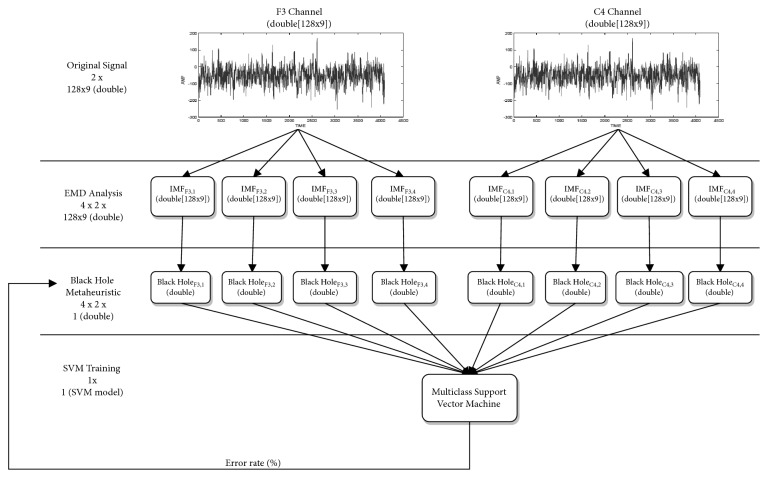
Proposed method using black hole algorithm.

Figure 5 illustrates the integration of the black hole algorithm into the process of creation of the classifier and its subsequent evaluation. The process is described in a loop way between the calculation of the predictability value and the performance evaluation of the created classifier. This approach allows improving the quality of the classifier, since it is used during the run of the algorithm itself.

## 4. Computational Experiments

After applying the approximation approach, we have analyzed the time complexity of the black hole algorithm into the process of creating the classifier and we illustrate that our proposal does not affect its performance. It can be determined that time complexity of the SampEn is given by *O*((*N*^2^/2)(1 − (1 − *r*)^*m*^)), where *N* represents the size of the array data and *m* is the number of matches and is much smaller than N. Finally, *r* represents the probability of two samples, *y*(*i*) and *y*(*j*) [69]. Now, by analyzing the approximate algorithm, it can be observed that the time complexity is given by *O*(*TN*), where *T* is a constant and represents the maximum number of iterations, while *N* is the size of population (stars). Although the incorporation of an optimization algorithm based on swarm intelligence can cause an increase in cyclomatic complexity (19 to 39) [70], this only affects the training phase. The classification phase, being subsequent to the search process of the best configuration of the SVM (gamma and C parameters), is not affected.

The performance of the black hole algorithm was experimentally evaluated by using a set of well-known validated signals using MAHNOB HCI Tagging Database [48].

The approximate approach has been implemented on the programming language C# and the experiments [71–73] have been executed on a 2.6 GHz Intel Core i7 with 16 GB RAM machine running Windows 7. The initial parameter setting used is detailed in Table 1.

Firstly, these parameter settings are adopted after a hard initial training phase, being the one that obtained the best results. Then, we considered previous works to compare the choice of parameter values as reported in [24].

A common method to recognize the emotion based on EEG signals uses the entropy factor to build the classifier. We have implemented this technique and the accuracy obtained was close to 84.77% producing an error of classification outperform to 15%. That can be attributed to the sample entropy that builds the classifier without iterating in order to find the best solution.

Towards the end of iterations, the approximate optimization method reaches an accuracy above to 93% illustrating again that its performance is better than sample entropy approach. All results are available in Appendix B.

**Figure 6:**
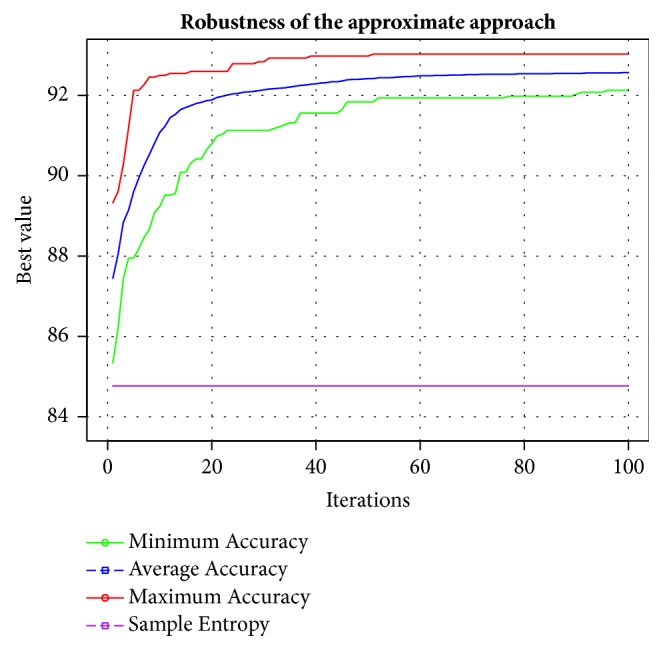
Convergence chart of the proposed method.

Figure 6 illustrates clearly the robustness of our proposed approach. Lower bound is given by the minimum accuracy found. If we only analyze this point only, we can see that immediately after the first iteration, the black hole algorithm always reaches a better value than found by the sample entropy method [42].

It is possible to conclude that the results are promising compared to those obtained with other SVM classifiers built by using the entropy factor. The proposed method used the MAHNOB HCI Tagging Database and reached a maximum accuracy level of 93.03%, with an average of 92.57%. Using the same dataset, a standard approach using the entropy factor to build a SVM classifier presents an average accuracy of 84.77%. More details can be seen in Appendix A and Appendix B.

This approach could be useful in emotion classification if the research goal would be to obtain relevant information in real time, for instance, incorporating an EEG in the classroom [74, 75]. This process would involve building a classifier for signal manipulation. The signal could be obtained online. Also, preprocessing techniques that have a high computational cost were not used, such as signal normalization or eye movement artifact cleaning using blind source separation. Apart from the computational cost, these techniques require a baseline signal previously recorded.

## 5. Conclusions and Future Works

Emotions have been subject to scientific research for more than a century, as they play many essential roles in people's lives [76]. In this paper, we have presented a new method based on an optimization approach for the building of an SVM classifier for EEG-emotion signals. This approach consists in applying the EMD method to decompose the signal. Then, sampled entropy is applied on the first 4 components. Next, with these initial characteristics, the black hole algorithm was used to optimize them and thus obtain the best combination of the SVM feature vectors to generate a higher accuracy.

EEG-emotion signals allow for the prediction and classification of data with automated noise reduction. The emotion research is especially complex due to the ecological paradigm requirement, specifically the trigger stimuli, and emotional response generates high rate of noise. A common method is detailed in the background section, using entropy as a more relevant element. Nevertheless, results are not what was expected, reaching 85% in accuracy only.

In order to improve these computational results, we conducted an approximate method inspired on the black hole phenomenon. This algorithm is proposed to analyze the performance of an SVM classifier, allowing the extension of emotion ecological paradigms with EEG data.

We have tested our technique using a validated emotion signal, named MAHNOB HCI Tagging Database. Results show that the optimization algorithm allows the SMV classifier to surpass 90% in accuracy in its first iterations, even reaching 93%; furthermore, it is highly competitive with those presented in the related works section.

Particularly, these results are compatible with those obtained with the EEG-emotion signal with wavelet entropy and Support Vector Machine classifier proposed by Çelikkanat, but with higher accuracy [6].

As future works, we believe that using new approximate optimization algorithms will allow us to find better results to compare the SVM classifier performance. Moreover, we intend to incorporate an autonomous version of these algorithms so that the self-adaptive of its parameters is not complex and suited to the instance of the problem, as described in [9, 11].

On the other hand, we propose an integration of autonomous search in the parameter settings process, in order to find the best values during the run. This research can lead towards new study lines.

## Figures and Tables

**Algorithm 1:**
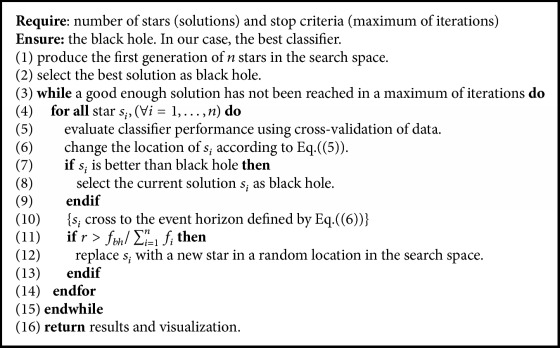
Black hole algorithm.

**Table 1:** Parameter setting to the entropy and the black hole algorithm.

**Section **	** Component **	** Description **	** Value **
Data selection	Number of sessions	Emotion elicitation trials	563
	Frequency	Each second has 128 samples or values	128 Hz
	Frame	To classify each frame it lasts 9 seconds, without overlapping	9 sec.

Sample entropy	*N*	Number of samples	128 samples
	*m*	Embedded dimension	2
	*r*	Probability of similarity on two simultaneous datasets	0.15

Empirical Mode Decomposition	Order	Number of IMFs	4

Black Hole Algorithm	*n*	Number of stars (solutions)	30
	*T*	Maximum iterations	100

Miscellaneous	–	Runs of the approximate approach	30
	–	Number of used cores (processors)	8

**Table 2:** Computational results of the approximate approach.

**It. **	**Accuracy**			
	Minimum	Average	Standard Deviation	Maximum
**1**	85.34	87.45	1.18E+04	89.33

**2**	86.20	88.05	8.89E+03	89.61

**3**	87.43	88.84	6.67E+03	90.28

**4**	87.95	89.14	7.32E+03	91.18

**5**	87.95	89.61	1.10E+04	92.13

**6**	88.19	89.95	1.15E+04	92.13

**7**	88.47	90.26	1.13E+04	92.27

**8**	88.66	90.53	1.15E+04	92.46

**9**	89.09	90.81	1.05E+04	92.46

**10**	89.23	91.08	9.90E+03	92.50

**11**	89.52	91.23	9.35E+03	92.50

**12**	89.52	91.45	7.81E+03	92.55

**13**	89.56	91.53	7.74E+03	92.55

**14**	90.09	91.65	6.72E+03	92.55

**15**	90.09	91.70	6.37E+03	92.55

**16**	90.32	91.75	5.98E+03	92.60

**17**	90.42	91.80	5.61E+03	92.60

**18**	90.42	91.83	5.58E+03	92.60

**19**	90.65	91.87	5.43E+03	92.60

**20**	90.80	91.89	5.28E+03	92.60

**Table 3:** Computational results of the approximate approach (continuation).

**It. **	**Accuracy**			
	Minimum	Average	Standard Deviation	Maximum
**21**	90.99	91.95	4.94E+03	92.60

**22**	91.03	91.98	4.64E+03	92.60

**23**	91.13	92.01	4.48E+03	92.60

**24**	91.13	92.04	4.37E+03	92.79

**25**	91.13	92.05	4.41E+03	92.79

**26**	91.13	92.08	4.35E+03	92.79

**27**	91.13	92.09	4.33E+03	92.79

**28**	91.13	92.10	4.27E+03	92.79

**29**	91.13	92.12	4.27E+03	92.84

**30**	91.13	92.14	4.17E+03	92.84

**31**	91.13	92.16	4.15E+03	92.93

**32**	91.18	92.17	4.13E+03	92.93

**33**	91.22	92.18	4.08E+03	92.93

**34**	91.27	92.19	4.12E+03	92.93

**35**	91.32	92.21	3.99E+03	92.93

**36**	91.32	92.23	3.91E+03	92.93

**37**	91.56	92.25	3.67E+03	92.93

**38**	91.56	92.26	3.53E+03	92.93

**39**	91.56	92.28	3.53E+03	92.98

**40**	91.56	92.29	3.52E+03	92.98

**Table 4:** Computational results of the approximate approach (continuation).

**It. **	** Accuracy **			
	Minimum	Average	Standard Deviation	Maximum
**41**	91.56	92.31	3.40E+03	92.98

**42**	91.56	92.32	3.29E+03	92.98

**43**	91.56	92.34	3.29E+03	92.98

**44**	91.56	92.34	3.27E+03	92.98

**45**	91.65	92.36	3.11E+03	92.98

**46**	91.84	92.39	2.78E+03	92.98

**47**	91.84	92.40	2.82E+03	92.98

**48**	91.84	92.40	2.81E+03	92.98

**49**	91.84	92.41	2.82E+03	92.98

**50**	91.84	92.42	2.84E+03	92.98

**51**	91.84	92.42	2.88E+03	93.03

**52**	91.94	92.44	2.72E+03	93.03

**53**	91.94	92.44	2.75E+03	93.03

**54**	91.94	92.44	2.73E+03	93.03

**55**	91.94	92.45	2.76E+03	93.03

**56**	91.94	92.46	2.84E+03	93.03

**57**	91.94	92.47	2.84E+03	93.03

**58**	91.94	92.47	2.79E+03	93.03

**59**	91.94	92.48	2.80E+03	93.03

**60 **	91.94	92.49	2.74E+03	93.03

**Table 5:** Computational results of the approximate approach (continuation).

**It. **	**Accuracy**			
	Minimum	Average	Standard Deviation	Maximum
**61**	91.94	92.49	2.74E+03	93.03

**62**	91.94	92.49	2.70E+03	93.03

**63**	91.94	92.50	2.68E+03	93.03

**64**	91.94	92.50	2.70E+03	93.03

**65**	91.94	92.50	2.70E+03	93.03

**66**	91.94	92.51	2.65E+03	93.03

**67**	91.94	92.51	2.60E+03	93.03

**68**	91.94	92.51	2.61E+03	93.03

**69**	91.94	92.52	2.60E+03	93.03

**70**	91.94	92.52	2.59E+03	93.03

**71**	91.94	92.52	2.59E+03	93.03

**72**	91.94	92.53	2.59E+03	93.03

**73**	91.94	92.53	2.59E+03	93.03

**74**	91.94	92.53	2.61E+03	93.03

**75**	91.94	92.53	2.62E+03	93.03

**76**	91.94	92.53	2.62E+03	93.03

**77**	91.98	92.53	2.59E+03	93.03

**78**	91.98	92.53	2.61E+03	93.03

**79**	91.98	92.54	2.58E+03	93.03

**80**	91.98	92.54	2.58E+03	93.03

**Table 6:** Computational results of the approximate approach (final).

**It. **	**Accuracy**			
	Minimum	Average	Standard Deviation	Maximum
**81**	91.98	92.54	2.59E+03	93.03

**82**	91.98	92.54	2.59E+03	93.03

**83**	91.98	92.54	2.59E+03	93.03

**84**	91.98	92.54	2.58E+03	93.03

**85**	91.98	92.54	2.55E+03	93.03

**86**	91.98	92.55	2.57E+03	93.03

**87**	91.98	92.55	2.55E+03	93.03

**88**	91.98	92.55	2.55E+03	93.03

**89**	91.98	92.55	2.55E+03	93.03

**90**	92.03	92.55	2.51E+03	93.03

**91**	92.08	92.55	2.48E+03	93.03

**92**	92.08	92.56	2.49E+03	93.03

**93**	92.08	92.56	2.49E+03	93.03

**94**	92.08	92.56	2.49E+03	93.03

**95**	92.08	92.56	2.49E+03	93.03

**96**	92.13	92.56	2.43E+03	93.03

**97**	92.13	92.56	2.43E+03	93.03

**98**	92.13	92.56	2.41E+03	93.03

**99**	92.13	92.57	2.41E+03	93.03

**100**	92.13	92.57	2.41E+03	93.03

**Table 7:** Dataset of experimental results. Twenty-five first iterations of the ten first runs.

**Iterations **	**Runs**									
	**#1**	**#2**	**#3**	**#4**	**#5**	**#6**	**#7**	**#8**	**#9**	**#10**
**1**	88.24	88.57	87.95	89.04	86.48	88.05	86.15	87.33	88.85	86.95

**2**	88.76	88.57	88.14	89.04	86.48	88.05	87.43	87.33	89.61	88.24

**3**	89.28	89.52	88.76	89.04	87.43	88.43	89.18	88.66	90.28	88.47

**4**	91.18	89.52	89.23	89.04	88.43	88.61	89.47	88.9	90.28	89.23

**5**	91.18	91.08	90.42	89.47	88.61	89.33	90.04	88.9	92.13	89.8

**6**	91.46	91.08	91.41	90.32	88.61	89.37	91.32	89.14	92.13	90.23

**7**	91.46	91.37	91.46	90.32	89.04	89.37	91.46	89.47	92.27	90.37

**8**	91.75	91.46	92.46	90.61	89.47	89.37	91.98	89.47	92.27	90.56

**9**	91.84	91.46	92.46	90.94	90.09	89.61	91.98	90.32	92.31	91.51

**10**	91.89	91.94	92.5	90.94	90.56	90.42	92.13	90.7	92.31	91.51

**11**	91.94	91.94	92.5	90.94	91.03	90.42	92.13	90.75	92.31	91.51

**12**	91.98	91.98	92.5	90.94	91.46	90.75	92.22	91.37	92.31	91.51

**13**	91.98	92.13	92.55	90.94	91.46	90.8	92.22	91.6	92.31	91.6

**14**	91.98	92.31	92.55	90.94	91.65	91.13	92.22	91.6	92.31	91.6

**15**	91.98	92.31	92.55	90.99	91.7	91.13	92.22	91.75	92.31	91.6

**16**	92.03	92.36	92.6	91.03	91.75	91.13	92.22	91.75	92.36	91.6

**17**	92.03	92.46	92.6	91.08	91.98	91.13	92.22	91.75	92.36	91.6

**18**	92.13	92.46	92.6	91.08	91.98	91.13	92.22	91.75	92.41	91.7

**19**	92.13	92.46	92.6	91.08	91.98	91.13	92.22	91.75	92.5	91.7

**20**	92.17	92.46	92.6	91.22	91.98	91.13	92.22	91.79	92.5	91.89

**21**	92.17	92.46	92.6	91.37	92.03	91.13	92.22	91.98	92.5	91.89

**22**	92.17	92.46	92.6	91.37	92.03	91.13	92.22	91.98	92.5	91.89

**23**	92.17	92.46	92.6	91.37	92.13	91.13	92.31	92.13	92.5	91.89

**24**	92.17	92.5	92.6	91.51	92.13	91.13	92.31	92.13	92.5	91.89

**25**	92.27	92.5	92.6	91.51	92.13	91.13	92.36	92.13	92.5	91.89

**Table 8:** Dataset of experimental results. Twenty-five second iterations of the ten first runs.

**Iterations**	**Runs**									
	**#1**	**#2**	**#3**	**#4**	**#5**	**#6**	**#7**	**#8**	**#9**	**#10**
**26**	92.27	92.5	92.6	91.51	92.31	91.13	92.41	92.13	92.5	91.89

**27**	92.27	92.5	92.6	91.51	92.31	91.13	92.46	92.13	92.5	91.89

**28**	92.27	92.5	92.6	91.51	92.31	91.18	92.55	92.13	92.5	91.89

**29**	92.27	92.5	92.6	91.56	92.46	91.18	92.55	92.13	92.5	91.89

**30**	92.27	92.5	92.6	91.56	92.46	91.18	92.65	92.13	92.5	91.94

**31**	92.27	92.5	92.6	91.56	92.46	91.27	92.65	92.17	92.5	91.94

**32**	92.27	92.5	92.6	91.56	92.5	91.27	92.65	92.22	92.5	91.98

**33**	92.27	92.5	92.6	91.56	92.5	91.27	92.65	92.27	92.5	91.98

**34**	92.27	92.5	92.6	91.56	92.65	91.27	92.69	92.41	92.5	92.03

**35**	92.27	92.5	92.6	91.56	92.79	91.32	92.69	92.41	92.5	92.13

**36**	92.27	92.5	92.6	91.56	92.88	91.32	92.69	92.41	92.5	92.17

**37**	92.27	92.5	92.6	91.56	92.88	91.6	92.69	92.41	92.5	92.17

**38**	92.27	92.5	92.6	91.56	92.88	91.6	92.69	92.41	92.5	92.17

**39**	92.27	92.5	92.6	91.56	92.98	91.6	92.69	92.41	92.5	92.17

**40**	92.27	92.5	92.6	91.56	92.98	91.6	92.69	92.41	92.5	92.27

**41**	92.27	92.5	92.6	91.56	92.98	91.6	92.69	92.41	92.5	92.36

**42**	92.27	92.5	92.6	91.56	92.98	91.65	92.69	92.41	92.5	92.5

**43**	92.27	92.5	92.6	91.56	92.98	91.65	92.69	92.41	92.5	92.5

**44**	92.27	92.5	92.6	91.56	92.98	91.65	92.69	92.41	92.5	92.5

**45**	92.27	92.5	92.6	91.7	92.98	91.65	92.69	92.41	92.5	92.5

**46**	92.27	92.5	92.6	91.89	92.98	91.84	92.69	92.41	92.5	92.5

**47**	92.27	92.5	92.6	91.89	92.98	91.84	92.69	92.41	92.5	92.5

**48**	92.27	92.5	92.6	91.89	92.98	91.84	92.69	92.41	92.5	92.5

**49**	92.27	92.5	92.6	91.89	92.98	91.84	92.69	92.41	92.5	92.5

**50**	92.31	92.5	92.6	91.94	92.98	91.84	92.69	92.41	92.5	92.55

**Table 9:** Dataset of experimental results. Twenty-five third iterations of the ten first runs.

**Iterations **	**Runs**									
	**#1**	**#2**	**#3**	**#4**	**#5**	**#6**	**#7**	**#8**	**#9**	**#10**
**51**	92.31	92.5	92.6	91.94	93.03	91.84	92.69	92.41	92.5	92.6

**52**	92.31	92.5	92.6	91.94	93.03	92.17	92.69	92.41	92.5	92.65

**53**	92.31	92.5	92.6	91.94	93.03	92.17	92.69	92.41	92.5	92.65

**54**	92.31	92.5	92.6	91.94	93.03	92.17	92.69	92.41	92.5	92.65

**55**	92.31	92.5	92.6	91.94	93.03	92.17	92.69	92.41	92.5	92.65

**56**	92.31	92.5	92.6	91.94	93.03	92.22	92.69	92.41	92.5	92.65

**57**	92.31	92.5	92.6	91.94	93.03	92.22	92.69	92.41	92.5	92.65

**58**	92.31	92.5	92.6	91.94	93.03	92.22	92.69	92.41	92.5	92.69

**59**	92.31	92.5	92.6	91.94	93.03	92.22	92.69	92.41	92.5	92.69

**60**	92.31	92.5	92.6	91.94	93.03	92.27	92.69	92.41	92.55	92.69

**61**	92.31	92.5	92.6	91.94	93.03	92.27	92.69	92.41	92.55	92.69

**62**	92.31	92.5	92.6	92.03	93.03	92.27	92.69	92.41	92.55	92.69

**63**	92.31	92.5	92.6	92.03	93.03	92.31	92.69	92.41	92.55	92.69

**64**	92.31	92.5	92.6	92.03	93.03	92.31	92.69	92.41	92.55	92.69

**65**	92.31	92.5	92.6	92.03	93.03	92.31	92.69	92.41	92.55	92.69

**66**	92.31	92.5	92.6	92.08	93.03	92.31	92.69	92.41	92.55	92.69

**67**	92.31	92.5	92.6	92.13	93.03	92.31	92.69	92.41	92.55	92.69

**68**	92.31	92.5	92.6	92.13	93.03	92.31	92.69	92.41	92.55	92.69

**69**	92.31	92.5	92.6	92.13	93.03	92.31	92.69	92.41	92.55	92.69

**70**	92.31	92.5	92.6	92.13	93.03	92.36	92.69	92.41	92.55	92.69

**71**	92.31	92.5	92.6	92.13	93.03	92.36	92.69	92.41	92.55	92.69

**72**	92.31	92.5	92.6	92.13	93.03	92.36	92.69	92.41	92.55	92.69

**73**	92.31	92.5	92.6	92.13	93.03	92.36	92.69	92.41	92.55	92.69

**74**	92.31	92.5	92.6	92.13	93.03	92.36	92.69	92.41	92.55	92.69

**75**	92.31	92.5	92.6	92.13	93.03	92.36	92.69	92.41	92.55	92.69

**Table 10:** Dataset of experimental results. Twenty-five fourth iterations of the ten first runs.

**Iterations **	**Runs**									
	**#1**	**#2**	**#3**	**#4**	**#5**	**#6**	**#7**	**#8**	**#9**	**#10**
**76**	92.31	92.5	92.6	92.13	93.03	92.36	92.69	92.41	92.55	92.69

**77**	92.31	92.5	92.6	92.13	93.03	92.36	92.69	92.41	92.55	92.69

**78**	92.31	92.5	92.6	92.13	93.03	92.36	92.69	92.41	92.55	92.69

**79**	92.31	92.5	92.6	92.13	93.03	92.41	92.69	92.41	92.55	92.69

**80**	92.31	92.5	92.6	92.13	93.03	92.41	92.69	92.41	92.55	92.69

**81**	92.31	92.5	92.6	92.13	93.03	92.41	92.69	92.41	92.55	92.69

**82**	92.31	92.5	92.6	92.13	93.03	92.46	92.69	92.41	92.55	92.69

**83**	92.31	92.5	92.6	92.13	93.03	92.46	92.69	92.41	92.55	92.69

**84**	92.31	92.5	92.6	92.13	93.03	92.46	92.69	92.46	92.55	92.69

**85**	92.31	92.5	92.6	92.13	93.03	92.46	92.69	92.46	92.55	92.69

**86**	92.31	92.5	92.6	92.13	93.03	92.46	92.69	92.46	92.55	92.69

**87**	92.41	92.5	92.6	92.13	93.03	92.46	92.69	92.46	92.55	92.69

**88**	92.41	92.5	92.6	92.13	93.03	92.46	92.69	92.46	92.55	92.69

**89**	92.41	92.5	92.6	92.13	93.03	92.46	92.69	92.46	92.55	92.69

**90**	92.41	92.5	92.6	92.13	93.03	92.46	92.69	92.46	92.55	92.69

**91**	92.41	92.5	92.6	92.13	93.03	92.46	92.69	92.46	92.55	92.69

**92**	92.41	92.5	92.6	92.13	93.03	92.46	92.69	92.46	92.55	92.69

**93**	92.41	92.5	92.6	92.13	93.03	92.46	92.69	92.46	92.55	92.69

**94**	92.41	92.5	92.6	92.13	93.03	92.46	92.69	92.46	92.55	92.69

**95**	92.41	92.5	92.6	92.13	93.03	92.46	92.69	92.46	92.55	92.69

**96**	92.41	92.5	92.6	92.13	93.03	92.46	92.69	92.46	92.55	92.69

**97**	92.41	92.5	92.6	92.13	93.03	92.46	92.69	92.46	92.55	92.69

**98**	92.41	92.5	92.6	92.13	93.03	92.46	92.69	92.46	92.55	92.69

**99**	92.41	92.5	92.6	92.13	93.03	92.46	92.69	92.46	92.55	92.69

**100**	92.41	92.5	92.6	92.13	93.03	92.46	92.69	92.46	92.55	

**Table 11:** Dataset of experimental results. Twenty-five first iterations of the ten second runs.

**Iterations **	**Runs**									
	**#11**	**#12**	**#13**	**#14**	**#15**	**#16**	**#17**	**#18**	**#19**	**#20**
**1**	86.86	87	85.91	87.86	88.52	88.28	88.43	85.39	86.39	86.2

**2**	87.57	88.28	88.24	87.86	88.52	88.28	88.52	86.48	87.9	86.2

**3**	88.57	88.95	88.43	89.71	88.52	89.14	88.71	88.9	88.09	87.95

**4**	88.85	88.95	88.8	89.75	88.66	89.14	88.8	88.9	88.57	87.95

**5**	89.14	89.71	88.8	90.09	89.28	89.14	88.8	88.9	89.42	87.95

**6**	89.28	90.51	88.8	90.09	90.51	89.94	88.8	89.33	90.37	88.19

**7**	89.52	90.51	89.42	91.94	91.37	90.04	89.28	89.33	90.51	88.47

**8**	89.71	90.8	89.42	91.94	91.37	90.18	89.37	89.33	91.27	90.04

**9**	89.94	90.84	89.42	92.13	91.37	90.94	89.9	89.33	91.27	90.56

**10**	90.37	91.08	89.52	92.46	91.79	91.84	89.9	89.42	91.27	90.89

**11**	90.56	91.18	89.52	92.46	91.79	91.84	90.04	89.52	91.75	90.89

**12**	91.27	91.51	89.56	92.55	91.79	91.84	91.22	89.52	91.84	90.89

**13**	91.27	91.56	89.56	92.55	91.79	91.89	91.22	89.66	91.94	90.89

**14**	91.27	91.7	90.56	92.55	91.84	91.89	91.7	90.09	92.13	90.89

**15**	91.37	91.75	91.13	92.55	91.84	91.89	91.7	90.09	92.13	90.99

**16**	91.51	91.75	91.13	92.55	91.84	91.89	91.7	90.42	92.13	90.99

**17**	91.6	91.79	91.18	92.55	91.84	91.89	91.79	90.42	92.13	90.99

**18**	91.6	91.79	91.18	92.55	91.89	91.89	91.79	90.42	92.13	90.99

**19**	91.7	91.79	91.37	92.55	92.08	91.94	91.79	90.65	92.13	90.99

**20**	91.7	91.79	91.37	92.6	92.08	91.94	91.79	90.8	92.17	90.99

**21**	91.75	91.79	91.6	92.6	92.08	91.94	91.89	91.03	92.17	90.99

**22**	91.79	91.84	91.6	92.6	92.08	91.94	91.94	91.56	92.17	91.03

**23**	91.79	91.84	91.65	92.6	92.22	91.94	91.94	91.56	92.17	91.27

**24**	91.79	91.94	91.65	92.6	92.22	91.94	91.94	91.6	92.17	91.41

**25**	91.79	91.94	91.65	92.6	92.22	91.94	91.94	91.6	92.17	91.41

**Table 12:** Dataset of experimental results. Twenty-five second iterations of the ten second runs.

**Iterations **	**Runs**									
	**#11**	**#12**	**#13**	**#14**	**#15**	**#16**	**#17**	**#18**	**#19**	**#20**
**26**	91.79	91.94	91.65	92.6	92.22	91.94	91.94	91.98	92.17	91.41

**27**	91.79	91.94	91.65	92.6	92.22	91.94	91.94	91.98	92.17	91.41

**28**	91.79	91.94	91.75	92.6	92.22	91.98	91.94	92.03	92.17	91.41

**29**	91.84	91.94	91.79	92.6	92.22	91.98	91.94	92.03	92.17	91.41

**30**	91.84	91.94	91.89	92.6	92.22	92.03	91.94	92.08	92.17	91.65

**31**	91.84	91.94	91.89	92.6	92.22	92.03	91.94	92.17	92.17	91.65

**32**	91.84	91.94	91.89	92.6	92.22	92.03	91.94	92.27	92.17	91.65

**33**	91.84	91.94	91.94	92.6	92.22	92.03	91.94	92.27	92.17	91.65

**34**	91.84	91.94	91.94	92.6	92.22	92.08	91.94	92.27	92.17	91.65

**35**	91.84	91.94	91.94	92.6	92.22	92.08	91.94	92.27	92.17	91.7

**36**	91.89	91.94	91.94	92.6	92.22	92.13	91.94	92.27	92.17	91.7

**37**	91.89	91.94	91.94	92.6	92.27	92.17	91.98	92.31	92.17	91.75

**38**	91.89	91.94	91.94	92.6	92.27	92.22	92.08	92.36	92.17	91.75

**39**	91.89	91.94	91.94	92.69	92.27	92.22	92.17	92.36	92.17	91.94

**40**	91.89	91.94	91.94	92.69	92.27	92.27	92.17	92.36	92.17	91.94

**41**	91.89	91.94	91.94	92.74	92.27	92.36	92.17	92.36	92.17	92.13

**42**	92.03	91.94	91.98	92.74	92.27	92.36	92.31	92.36	92.17	92.13

**43**	92.03	91.94	91.98	92.74	92.27	92.36	92.41	92.36	92.17	92.17

**44**	92.08	91.94	91.98	92.74	92.27	92.36	92.5	92.36	92.17	92.17

**45**	92.08	91.94	92.03	92.74	92.27	92.36	92.5	92.36	92.17	92.27

**46**	92.27	91.94	92.03	92.74	92.27	92.36	92.5	92.36	92.22	92.46

**47**	92.27	91.94	92.03	92.84	92.27	92.36	92.5	92.36	92.22	92.55

**48**	92.27	91.94	92.03	92.84	92.27	92.41	92.5	92.36	92.31	92.55

**49**	92.27	91.94	92.03	92.84	92.27	92.41	92.5	92.36	92.31	92.6

**50**	92.27	91.94	92.03	92.84	92.27	92.46	92.5	92.36	92.36	92.79

**Table 13:** Dataset of experimental results. Twenty-five third iterations of the ten second runs.

**Iterations **	**Runs**									
	**#11**	**#12**	**#13**	**#14**	**#15**	**#16**	**#17**	**#18**	**#19**	**#20**
**51**	92.27	91.94	92.03	92.84	92.27	92.46	92.5	92.36	92.36	92.79

**52**	92.27	91.94	92.03	92.84	92.27	92.5	92.5	92.36	92.36	92.79

**53**	92.27	91.94	92.03	92.84	92.27	92.5	92.5	92.36	92.46	92.84

**54**	92.27	91.94	92.08	92.84	92.27	92.5	92.5	92.36	92.5	92.84

**55**	92.31	91.94	92.08	92.84	92.27	92.55	92.5	92.36	92.69	92.84

**56**	92.31	91.94	92.08	92.88	92.27	92.55	92.5	92.36	92.74	92.93

**57**	92.31	91.94	92.13	92.93	92.27	92.55	92.5	92.41	92.74	92.93

**58**	92.36	91.94	92.13	92.93	92.27	92.55	92.5	92.41	92.74	92.93

**59**	92.36	91.94	92.13	92.93	92.27	92.55	92.5	92.41	92.79	92.93

**60**	92.36	91.94	92.13	92.93	92.27	92.55	92.5	92.41	92.79	92.93

**61**	92.36	91.94	92.13	92.93	92.27	92.55	92.5	92.41	92.79	92.93

**62**	92.36	91.94	92.13	92.93	92.27	92.55	92.5	92.41	92.84	92.93

**63**	92.41	91.94	92.13	92.93	92.27	92.55	92.5	92.41	92.84	92.93

**64**	92.41	91.94	92.13	92.93	92.27	92.55	92.5	92.41	92.88	92.93

**65**	92.41	91.94	92.13	92.93	92.27	92.55	92.5	92.41	92.88	92.93

**66**	92.41	91.94	92.13	92.93	92.27	92.55	92.5	92.46	92.88	92.93

**67**	92.41	91.94	92.13	92.93	92.27	92.55	92.5	92.46	92.88	92.93

**68**	92.41	91.94	92.13	92.93	92.27	92.55	92.5	92.46	92.88	92.93

**69**	92.41	91.94	92.13	92.93	92.27	92.55	92.5	92.5	92.88	92.93

**70**	92.41	91.94	92.13	92.93	92.27	92.55	92.5	92.5	92.88	92.93

**71**	92.41	91.94	92.13	92.93	92.27	92.55	92.5	92.55	92.88	92.93

**72**	92.41	91.94	92.13	92.93	92.27	92.55	92.5	92.55	92.88	92.93

**73**	92.41	91.94	92.13	92.93	92.27	92.55	92.5	92.55	92.88	92.93

**74**	92.41	91.94	92.13	92.93	92.27	92.55	92.5	92.55	92.88	92.93

**75**	92.41	91.94	92.13	92.93	92.27	92.55	92.5	92.55	92.88	92.93

**Table 14:** Dataset of experimental results. Twenty-five fourth iterations of the ten second runs.

**Iterations **	**Runs**									
	**#11**	**#12**	**#13**	**#14**	**#15**	**#16**	**#17**	**#18**	**#19**	**#20**
**76**	92.41	91.94	92.13	92.93	92.27	92.55	92.5	92.55	92.88	92.93

**77**	92.41	91.98	92.13	92.93	92.27	92.55	92.5	92.55	92.88	92.93

**78**	92.41	91.98	92.13	92.93	92.27	92.55	92.5	92.55	92.88	92.93

**79**	92.41	91.98	92.13	92.93	92.27	92.55	92.5	92.55	92.88	92.93

**80**	92.41	91.98	92.13	92.93	92.27	92.55	92.5	92.55	92.88	92.93

**81**	92.41	91.98	92.13	92.93	92.27	92.55	92.5	92.55	92.88	92.93

**82**	92.41	91.98	92.13	92.93	92.27	92.55	92.5	92.55	92.88	92.93

**83**	92.41	91.98	92.13	92.93	92.27	92.55	92.5	92.55	92.88	92.93

**84**	92.41	91.98	92.13	92.93	92.27	92.55	92.5	92.55	92.88	92.93

**85**	92.41	91.98	92.17	92.93	92.27	92.55	92.5	92.55	92.88	92.93

**86**	92.46	91.98	92.17	92.93	92.27	92.55	92.5	92.55	92.93	92.93

**87**	92.46	91.98	92.17	92.93	92.27	92.55	92.5	92.55	92.93	92.93

**88**	92.46	91.98	92.17	92.93	92.27	92.55	92.5	92.55	92.93	92.93

**89**	92.46	91.98	92.17	92.93	92.27	92.55	92.5	92.55	92.93	92.93

**90**	92.46	92.03	92.17	92.93	92.27	92.55	92.5	92.55	92.93	92.93

**91**	92.46	92.08	92.17	92.93	92.27	92.55	92.5	92.55	92.93	92.93

**92**	92.46	92.08	92.17	92.93	92.27	92.55	92.5	92.65	92.93	92.93

**93**	92.46	92.08	92.17	92.93	92.27	92.55	92.5	92.65	92.93	92.93

**94**	92.46	92.08	92.17	92.93	92.27	92.55	92.5	92.65	92.93	92.93

**95**	92.46	92.08	92.17	92.93	92.27	92.55	92.5	92.65	92.93	92.93

**96**	92.46	92.17	92.17	92.93	92.31	92.55	92.5	92.65	92.93	92.93

**97**	92.46	92.17	92.17	92.93	92.31	92.55	92.5	92.65	92.93	92.93

**98**	92.46	92.17	92.17	92.93	92.36	92.55	92.5	92.65	92.93	92.93

**99**	92.46	92.17	92.17	92.93	92.41	92.55	92.5	92.65	92.93	92.93

**100**	92.46	92.17	92.17	92.93	92.41	92.55	92.5	92.65	92.93	92.93

**Table 15:** Dataset of experimental results. Twenty-five first iterations of the ten third runs.

**Iterations **	**Runs**									
	**#21**	**#22**	**#23**	**#24**	**#25**	**#26**	**#27**	**#28**	**#29**	**#30**
**1**	85.34	87.81	89.33	88.14	87.95	87.76	88.85	88.66	86.2	86.39

**2**	86.67	88.95	89.33	88.14	87.95	87.76	89.61	88.66	87.81	88.33

**3**	88.14	88.95	89.71	88.61	87.95	89.66	90.28	88.66	88.8	88.61

**4**	88.47	88.95	90.51	90.04	88.33	89.66	90.28	88.8	88.85	88.66

**5**	88.61	88.95	91.94	90.04	88.52	89.66	92.13	88.85	89.28	88.8

**6**	88.66	88.95	91.98	90.04	88.61	90.32	92.13	88.9	89.37	88.99

**7**	88.66	89.04	92.03	90.56	89.47	90.32	92.27	89.47	90.23	88.99

**8**	88.66	89.33	92.17	91.18	89.75	90.56	92.27	89.47	90.84	88.99

**9**	89.47	89.66	92.17	91.89	90.04	91.46	92.31	89.75	91.13	89.09

**10**	89.47	90.04	92.17	91.89	91.03	91.46	92.31	90.32	91.13	89.23

**11**	89.61	91.6	92.17	92.13	91.08	91.46	92.31	90.42	91.7	89.56

**12**	90.89	91.6	92.17	92.17	91.13	91.46	92.31	90.89	91.84	90.09

**13**	90.89	91.84	92.17	92.31	91.32	91.6	92.31	91.79	91.84	90.09

**14**	90.89	92.08	92.22	92.31	91.32	91.6	92.31	91.98	91.84	90.09

**15**	90.99	92.08	92.22	92.36	91.37	91.79	92.31	91.98	91.89	90.09

**16**	90.99	92.08	92.22	92.36	91.46	91.98	92.36	92.17	91.94	90.32

**17**	91.13	92.27	92.22	92.36	91.46	91.98	92.36	92.17	91.94	90.8

**18**	91.13	92.27	92.22	92.36	91.46	92.27	92.41	92.17	91.94	91.03

**19**	91.13	92.46	92.22	92.36	91.46	92.27	92.5	92.22	91.94	91.08

**20**	91.13	92.46	92.22	92.36	91.56	92.27	92.5	92.27	91.94	91.08

**21**	91.13	92.5	92.22	92.36	92.03	92.27	92.5	92.27	91.94	91.08

**22**	91.13	92.55	92.22	92.36	92.03	92.27	92.5	92.31	91.94	91.13

**23**	91.13	92.6	92.22	92.41	92.08	92.27	92.5	92.31	91.94	91.27

**24**	91.13	92.79	92.27	92.46	92.08	92.27	92.5	92.31	91.94	91.41

**25**	91.13	92.79	92.27	92.46	92.17	92.27	92.5	92.36	91.94	91.41

**Table 16:** Dataset of experimental results. Twenty-five second iterations of the ten third runs.

**Iterations **	**Runs**									
	**#21**	**#22**	**#23**	**#24**	**#25**	**#26**	**#27**	**#28**	**#29**	**#30**
**26**	91.13	92.79	92.31	92.5	92.17	92.27	92.5	92.36	91.94	91.51

**27**	91.13	92.79	92.31	92.5	92.17	92.27	92.5	92.46	91.94	91.65

**28**	91.13	92.79	92.31	92.5	92.17	92.27	92.5	92.46	91.94	91.7

**29**	91.13	92.84	92.31	92.5	92.17	92.27	92.5	92.5	91.94	91.79

**30**	91.13	92.84	92.31	92.5	92.31	92.31	92.5	92.5	91.98	91.79

**31**	91.13	92.93	92.36	92.5	92.36	92.36	92.5	92.5	91.98	91.84

**32**	91.18	92.93	92.36	92.5	92.36	92.36	92.5	92.5	91.98	91.84

**33**	91.22	92.93	92.36	92.5	92.41	92.36	92.5	92.5	91.98	91.84

**34**	91.27	92.93	92.36	92.5	92.46	92.36	92.5	92.5	91.98	91.89

**35**	91.41	92.93	92.36	92.5	92.46	92.36	92.5	92.5	91.98	91.98

**36**	91.56	92.93	92.36	92.5	92.5	92.36	92.5	92.5	91.98	92.08

**37**	91.56	92.93	92.36	92.5	92.5	92.36	92.5	92.5	91.98	92.13

**38**	91.75	92.93	92.36	92.5	92.5	92.36	92.5	92.5	91.98	92.13

**39**	91.75	92.93	92.36	92.5	92.5	92.36	92.5	92.55	92.08	92.13

**40**	91.75	92.93	92.36	92.5	92.5	92.36	92.5	92.55	92.08	92.17

**41**	91.89	92.93	92.36	92.5	92.5	92.36	92.5	92.55	92.13	92.17

**42**	91.89	92.93	92.36	92.5	92.5	92.36	92.5	92.55	92.13	92.17

**43**	91.89	92.93	92.36	92.5	92.5	92.36	92.5	92.55	92.17	92.17

**44**	91.89	92.93	92.36	92.5	92.5	92.36	92.5	92.55	92.27	92.17

**45**	91.94	92.93	92.36	92.5	92.5	92.41	92.5	92.55	92.36	92.17

**46**	91.94	92.93	92.36	92.5	92.5	92.41	92.5	92.55	92.41	92.17

**47**	91.98	92.93	92.36	92.5	92.5	92.41	92.5	92.55	92.41	92.17

**48**	91.98	92.93	92.36	92.5	92.5	92.41	92.5	92.55	92.5	92.17

**49**	91.98	92.93	92.36	92.5	92.5	92.41	92.5	92.55	92.5	92.17

**50**	91.98	92.93	92.36	92.5	92.5	92.41	92.5	92.55	92.5	92.17

**Table 17:** Dataset of experimental results. Twenty-five third iterations of the ten third runs.

**Iterations **	**Runs**									
	**#21**	**#22**	**#23**	**#24**	**#25**	**#26**	**#27**	**#28**	**#29**	**#30**
**51**	91.98	92.93	92.36	92.5	92.5	92.41	92.5	92.55	92.5	92.17

**52**	91.98	92.93	92.36	92.5	92.5	92.41	92.5	92.55	92.5	92.17

**53**	91.98	92.93	92.36	92.5	92.5	92.41	92.5	92.55	92.5	92.17

**54**	91.98	92.93	92.36	92.5	92.5	92.41	92.5	92.55	92.5	92.17

**55**	91.98	92.93	92.36	92.5	92.5	92.41	92.5	92.55	92.5	92.17

**56**	91.98	92.93	92.36	92.5	92.5	92.41	92.5	92.55	92.5	92.17

**57**	91.98	92.93	92.36	92.5	92.5	92.41	92.5	92.55	92.5	92.17

**58**	92.03	92.93	92.36	92.5	92.5	92.41	92.5	92.55	92.5	92.22

**59**	92.03	92.93	92.36	92.5	92.5	92.41	92.5	92.55	92.5	92.27

**60**	92.08	92.93	92.36	92.5	92.5	92.41	92.55	92.55	92.5	92.41

**61**	92.08	92.93	92.36	92.5	92.5	92.41	92.55	92.55	92.5	92.41

**62**	92.08	92.93	92.36	92.5	92.5	92.41	92.55	92.55	92.5	92.41

**63**	92.08	92.93	92.36	92.5	92.5	92.41	92.55	92.55	92.5	92.41

**64**	92.08	92.93	92.36	92.5	92.5	92.41	92.55	92.55	92.5	92.41

**65**	92.08	92.93	92.36	92.5	92.5	92.41	92.55	92.55	92.5	92.41

**66**	92.13	92.93	92.36	92.5	92.5	92.41	92.55	92.55	92.5	92.6

**67**	92.17	92.93	92.36	92.5	92.55	92.41	92.55	92.55	92.5	92.6

**68**	92.17	92.93	92.36	92.5	92.55	92.41	92.55	92.55	92.5	92.65

**69**	92.22	92.93	92.36	92.5	92.6	92.41	92.55	92.55	92.5	92.69

**70**	92.22	92.93	92.36	92.5	92.6	92.41	92.55	92.55	92.5	92.69

**71**	92.22	92.93	92.36	92.5	92.6	92.41	92.55	92.55	92.5	92.69

**72**	92.22	92.93	92.36	92.5	92.65	92.41	92.55	92.55	92.5	92.69

**73**	92.22	92.93	92.36	92.5	92.65	92.41	92.55	92.55	92.5	92.69

**74**	92.22	92.93	92.36	92.5	92.65	92.41	92.55	92.55	92.5	92.74

**75**	92.22	92.93	92.36	92.5	92.65	92.41	92.55	92.55	92.5	92.79

**Table 18:** Dataset of experimental results. Twenty-five fourth iterations of the ten third runs.

**Iterations **	**Runs**									
	**#21**	**#22**	**#23**	**#24**	**#25**	**#26**	**#27**	**#28**	**#29**	**#30**
**76**	92.22	92.93	92.36	92.5	92.65	92.41	92.55	92.55	92.5	92.79

**77**	92.22	92.93	92.36	92.5	92.65	92.41	92.55	92.55	92.5	92.79

**78**	92.22	92.93	92.36	92.5	92.74	92.41	92.55	92.55	92.5	92.79

**79**	92.27	92.93	92.36	92.5	92.74	92.41	92.55	92.55	92.5	92.79

**80**	92.27	92.93	92.36	92.5	92.74	92.41	92.55	92.55	92.5	92.79

**81**	92.27	92.93	92.36	92.5	92.79	92.41	92.55	92.55	92.5	92.79

**82**	92.27	92.93	92.36	92.5	92.79	92.41	92.55	92.55	92.5	92.79

**83**	92.27	92.93	92.36	92.5	92.79	92.41	92.55	92.55	92.5	92.79

**84**	92.27	92.93	92.36	92.5	92.79	92.41	92.55	92.55	92.5	92.79

**85**	92.27	92.93	92.36	92.5	92.79	92.41	92.55	92.55	92.5	92.79

**86**	92.27	92.93	92.36	92.5	92.79	92.41	92.55	92.55	92.5	92.79

**87**	92.27	92.93	92.36	92.5	92.79	92.41	92.55	92.55	92.5	92.79

**88**	92.27	92.93	92.36	92.5	92.79	92.41	92.55	92.55	92.5	92.79

**89**	92.27	92.93	92.36	92.5	92.79	92.41	92.55	92.55	92.5	92.79

**90**	92.27	92.93	92.36	92.5	92.79	92.41	92.55	92.55	92.5	92.79

**91**	92.27	92.93	92.36	92.5	92.79	92.41	92.55	92.55	92.5	92.79

**92**	92.27	92.93	92.36	92.5	92.79	92.41	92.55	92.55	92.5	92.79

**93**	92.27	92.93	92.36	92.5	92.79	92.41	92.55	92.55	92.5	92.79

**94**	92.27	92.93	92.36	92.5	92.79	92.41	92.55	92.55	92.5	92.79

**95**	92.27	92.93	92.36	92.5	92.79	92.41	92.55	92.55	92.5	92.79

**96**	92.27	92.93	92.36	92.5	92.84	92.41	92.55	92.55	92.5	92.79

**97**	92.27	92.93	92.36	92.5	92.84	92.41	92.55	92.55	92.5	92.79

**98**	92.27	92.93	92.36	92.5	92.84	92.41	92.55	92.55	92.5	92.79

**99**	92.27	92.93	92.36	92.55	92.84	92.41	92.55	92.55	92.5	92.84

**100**	92.27	92.93	92.36	92.55	92.84	92.41	92.55	92.55	92.5	92.84

## Data Availability

The software developed and the data generated to support the findings of this study have been deposited in the Figshare repository (10.6084/m9.figshare.5588896, 10.6084/m9.figshare.5588911, and 10.6084/m9.figshare.5590000.v2).

## Appendix

### A. Summary of the Experimental Results

In Tables 2, 3, 4, 5, and 6, we show a summary of the computational results generated by using the approximate approach. All computational results can be seen in Appendix B. Executing the experiments, we can observe that the performance of the optimization algorithm to find the best values for building the SVM classifier was outperforming to entropy approach. If we analyze the resolution process, we can see that in the first ten iterations the black hole algorithm reaches a minimum and average accuracy close to 90%. Finally, the best value achieved is higher than 92%. In next iterations, the robustness of algorithm is demonstrated, according to the standard deviation values decrease as iterations occur.

### B. Details of the Experimental Results

In Tables 7, 8, 9, 10, 11, 12, 13, 14, 15, 16, 17, and 18 we illustrate all computational results that we allow analyzing the performance of the proposed mixed approach. These tables have the same headers, which are described below: column 1 (Iterations) corresponds to the identifier assigned to each iteration. Columns 2-11 (Runs) describe runs each iteration; i.e., for instance, in row 10 (iteration 10) and column six (run #5), of Table 7, we can see that our approach reaches an accuracy of 90.56%. The same description can be used for the other tables.
